# The combined impact of neutrophil-percentage-to-albumin ratio and depressive symptoms on mortality in US arthritis patients: insights from NHANES (2005–2018)

**DOI:** 10.3389/fpubh.2025.1545250

**Published:** 2025-03-06

**Authors:** Jinyue Bai, Taihong Lv, Hanming Yu, Zishuo Ji, Xiu Gu, Yun Gao, Li Ma

**Affiliations:** ^1^Department of General Practice, Aerospace Center Hospital, Beijing, China; ^2^Department of General Medicine, Beijing TianTan Hospital, Capital Medical University, Beijing, China; ^3^Department of Pulmonary and Critical Care Medicine, The Fourth Affiliated Hospital of China Medical University, Shenyang, China; ^4^Department of Neurology, Beijing TianTan Hospital, Capital Medical University, Beijing, China

**Keywords:** neutrophil-to-albumin ratio, arthritis, National Health and Nutrition Examination Survey, all-cause mortality, depression

## Abstract

**Background:**

The neutrophil-to-albumin ratio (NPAR) reflects inflammation and nutritional status, while depression significantly impacts survival in chronic disease patients. This study examines the independent and combined effects of NPAR and depressive symptoms on all-cause and cardiovascular mortality in arthritis patients.

**Methods:**

We analyzed a nationally representative sample of people with arthritisaged 40 and older from NHANES (2005–2018). NPAR assessed inflammation and nutritional status, while depressive symptoms were measured by PHQ-9. Weighted Cox regression examined the independent and joint associations of NPAR and PHQ-9 with all-cause and cardiovascular disease (CVD) mortality.

**Results:**

Our analysis indicated that higher NPAR levels combined with lower depressive symptoms (PHQ-9 < 10) significantly increased all-cause and CVD mortality risks in arthritis patients. In this group, the hazard ratio (HR) for all-cause mortality was 2.087, with a similarly elevated CVD mortality risk (HR = 2.614), underscoring NPAR’s predictive strength in non-depressed individuals. Among those with higher depressive symptoms, while elevated NPAR was still associated with increased mortality, its impact on CVD mortality was less marked, highlighting the need for further research into the NPAR-depression interaction.

**Conclusion:**

This study identifies NPAR as a key predictor of mortality in arthritis patients, particularly those with fewer depressive symptoms. NPAR significantly predicts all-cause and CVD mortality, underscoring its value as an inflammation and nutrition biomarker. Integrating NPAR in clinical practice could enhance individualized risk assessment and intervention for arthritis patients.

## 1 Introduction

Arthritis encompasses a range of joint diseases characterized by inflammation, commonly resulting in pain and progressive joint degeneration (1). Among these, rheumatoid arthritis (RA) and osteoarthritis (OA) are the most prevalent. Although the regulatory mechanisms underlying inflammation in RA and OA may differ, the excessive accumulation of catabolic factors, cytokines, and inflammatory cells in both conditions contributes significantly to the disruption of cartilage and bone homeostasis. At the cellular level, this imbalance manifests primarily as a dysregulation between osteoblast and osteoclast activity, excessive proliferation of synovial cells, and immune cell dysfunction. These pathophysiological alterations play a crucial role in the progression of joint damage and the exacerbation of disease symptoms in both RA and OA (2, 3). In 2020, an estimated 17,600 people worldwide had RA, with a global age-standardized prevalence rate of 208.8 cases per 100,000, reflecting a 14.1% increase since 1990 (4). Additionally, approximately 595 million people, or 7.6% of the global population, suffer from OA, with cases rising by 132.2% since 1990 (5). Without timely intervention, arthritis can lead to persistent pain, functional impairment, and reduced life expectancy (6, 7).

Over the past two decades, the prevalence of depression in the general population has been approximately 6%; however, among RA patients, the rate is notably higher at 16.8% (8). A longitudinal cohort study found that individuals with multi-site, hip, or knee OA were more likely to experience depressive symptoms compared to those without the disease (9). Recent studies also suggest depression plays a mediating role in the link between OA and cardiovascular mortality, accounting for 5.61% of this association (10). Similarly, research by La et al. indicated that depression mediates the relationship between RA and an increased risk of both all-cause and cardiovascular mortality (11).

Beyond the influence of depression, nutritional and inflammatory factors also significantly impact survival outcomes in arthritis (12, 13). The neutrophil-to-albumin ratio (NPAR), a blood biomarker reflecting the ratio of neutrophils to serum albumin levels, is an indicator of inflammatory status, infection, and nutritional state (14). NPAR’s dual role in assessing inflammation and nutrition has drawn attention for its prognostic value in inflammatory-related conditions such as diabetes (15), colorectal cancer (16), and acute kidney injury (17).

Several interventions have been shown to effectively mitigate the impact of these factors on arthritis outcomes. For example, early and continuous pharmacological treatments, including disease-modifying antirheumatic drugs (DMARDs) for RA and nonsteroidal anti-inflammatory drugs (NSAIDs) for OA, are crucial in controlling inflammation and preventing further joint damage (18, 19). Additionally, physical therapy and lifestyle interventions, such as exercise and weight management, can improve joint function, reduce pain, and alleviate depressive symptoms (20). Psychosocial interventions, such as cognitive-behavioral therapy (CBT) and antidepressant treatment, have demonstrated effectiveness in managing depression and its associated risks in people with arthritis (21). Nutritional interventions focusing on anti-inflammatory diets have also been shown to positively influence both disease activity and overall well-being in people with arthritis (22).

The National Health and Nutrition Examination Survey (NHANES) is a nationally representative, multi-stage survey utilizing standardized laboratory protocols and complex statistical methods (23). Using NHANES data, the primary objective of this study is to assess whether NPAR and depression are associated with mortality risk in arthritis patients. Furthermore, we evaluate the combined effects of NPAR and depressive status on mortality risk, aiming to shed light on determinants of long-term outcomes in people with arthritisand enhance clinical management approaches for this population.

## 2 Materials and methods

### 2.1 Data source and participants

Our analysis utilized data from NHANES, a stratified, multistage probability survey designed to represent the non-institutionalized civilian population of the United States. NHANES protocols received approval from the Institutional Review Board (IRB) of the National Center for Health Statistics, part of the Centers for Disease Control and Prevention (CDC). Informed consent was obtained from all participants prior to their inclusion in the study. Detailed protocols and consent forms are available online at https://www.cdc.gov/nchs/nhanes/.

**Figure 1:**
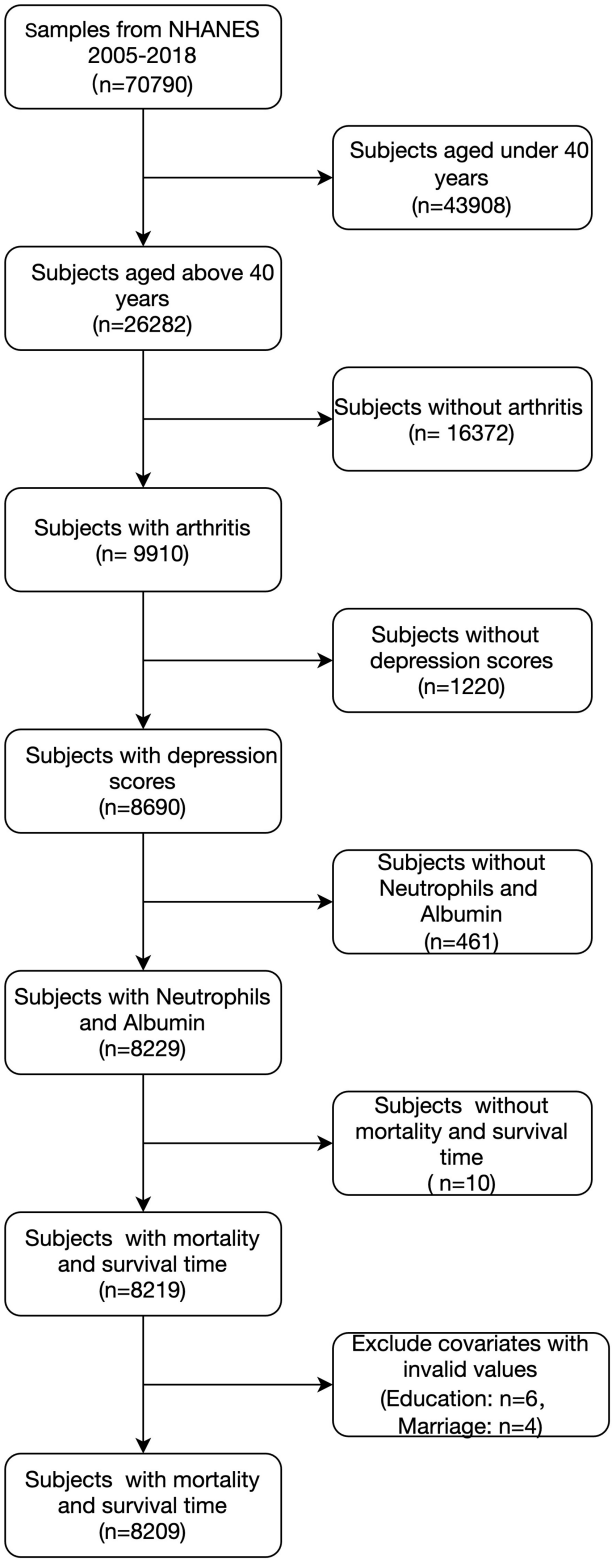
Flowchart of the population included in our final analysis.

Figure 1 outlines the study design, sampling process, and exclusion criteria applied. Data from seven NHANES cycles (2005–2018) were linked to mortality outcomes. Among the initial 70,790 participants, we excluded: (1) individuals under 40 years of age (*n* = 43,908), (2) people without arthritis (*n* = 16,372), (3) participants without depression questionnaire data (n = 1,220), (4) those without mortality or survival data (*n* = 10), and (5) participants missing relevant covariate information (*n* = 10). This resulted in a final sample of 8,209 participants for the analysis.

### 2.2 Assessment of neutrophil-percentage-to-albumin ratio

NPAR was calculated using the blood sample and the following formula: Neutrophil percentage (in total WBC count) (%) × 100/Albumin (g/dL).

### 2.3 Assessment of depressive symptoms

The Patient Health Questionnaire-9 (PHQ-9) was employed to assess depressive symptoms (24). PHQ-9 scores range from 0 to 27, with higher scores indicating greater severity of depressive symptoms (25). Based on PHQ-9 scores, participants were categorized into three groups: 0–4 (no depression), 5–9 (mild depression), and ≥ 10 (moderate to severe depression). Compared to clinical interview criteria, a PHQ-9 score of ≥10 demonstrates a sensitivity of 85% and a specificity of 89% for diagnosing major depressive disorder (MDD) (26).

### 2.4 Covariates

Based on previously published research, potential covariates associated with arthritis have been identified (27, 28). These covariates encompass socio-demographic elements, behavioral traits, and health characteristics.

The sociodemographic variables included gender (male or female), race and ethnicity (non-Hispanic White, non-Hispanic Black, Mexican American, other), educational attainment (Below high school graduate, High school graduate and above), marital status (Married, Widowed, Divorced, Separated, Never married, and Living with partner) and family poverty income ratio (< 1, ≥ 1).

Behavioral characteristics were delineated by sleep duration (< 7 h, 7 to <9, ≥ 9 h) and smoking status (never, former, current). Health-related factors encompassed body mass index (underweight/normal, overweight, and obese), presence of hypertension (no or yes), and diabetes status (no or yes).

Health-related factors encompassed BMI was categorized into 3 groups (<25, 25.0–29.9, ≥30). Diabetes was identified based on self-reported diagnosis, current use of insulin or antidiabetic medications, or by meeting one of the following thresholds: fasting plasma glucose ≥7.0 mmol/L, 2-h postprandial plasma glucose ≥200 mg/dL, or glycated hemoglobin A1c ≥ 6.5%. Hypertension was defined by self-reported diagnosis, current antihypertensive treatment, or systolic/diastolic blood pressure ≥ 140/90 mmHg. Cardiovascular disease (CVD) was classified based on a self-reported diagnosis of any of the following: stroke, angina, myocardial infarction, coronary heart disease, or heart failure.

### 2.5 Statistical analysis

In line with NHANES analytical guidelines, this study employed sample weighting, clustering, and stratification to estimate variance and ensure national representation of people with arthritis in the U.S. Baseline characteristics were reported across varying levels of NPAR and depressive symptoms, with continuous variables presented as mean ± standard error (SE) and categorical variables as percentages. Multivariable Cox proportional hazards regression models were applied to calculate hazard ratios (HRs) and 95% confidence intervals (CIs) for NPAR and/or depressive symptoms in relation to total and cardiovascular mortality. In the model 2, adjustments were made for age, sex, race/ethnicity, BMI (for PHQ-9 scores), poverty-to-income ratio, education, sleep duration, smoking, and relevant health factors (hypertension, diabetes, and cardiovascular disease history). Participants were divided into two groups—NPAR and depression—and multivariable Cox models were used to assess mortality risk, adjusting for the same covariates. Schoenfeld residuals were used to test the proportional risk hypothesis and no violations were observed. To reduce the risk of reverse causality, sensitivity analyses excluded deaths within the first 2 years of follow-up (29).

All analyses were performed using R software 4.2.3. A two-sided *p* value of less than 0.05 was considered statistically significant.

## 3 Results

### 3.1 Baseline characteristics

Table 1 presents the characteristics of people with arthritis stratified by NPAR levels. The findings reveal significant differences across NPAR groups in terms of age, race, BMI, depressive symptoms, diabetes, hypertension, CVD, and sleep duration. Patients in the high NPAR group had a median age of 63 years, a greater proportion of non-Hispanic White individuals (81%), a higher prevalence of obesity (56% with BMI ≥ 30), and a higher incidence of severe depressive symptoms (14% with PHQ-9 score ≥ 10). Additionally, the high NPAR group had increased rates of diabetes (31%), hypertension (68%), and CVD (25%) compared to other groups. Regarding sleep patterns, the high NPAR group showed a higher percentage of individuals sleeping ≥9 h (7.7%) and a lower percentage sleeping 7 to 9 h (58%). There were no significant differences across NPAR groups in gender distribution or smoking status. Overall, elevated NPAR levels were associated with older age, higher BMI, greater prevalence of chronic conditions like diabetes and CVD, and more severe depressive symptoms.

**Table 1:** Baseline characteristics.

Characteristic	Overall *N* = 46,498,743	NPAR~low *N* = 15,130,659	NPAR~intermediate *N* = 15,644,940	NPAR~high *N* = 15,723,144	*p*-value
Age (years)	62.0 (54.0, 71.0)	61.0 (53.0, 69.0)	62.0 (54.0, 70.0)	63.0 (54.0, 74.0)	<0.001
Gender					0.3
Male	3,399 (40%)	1,071 (40%)	1,149 (42%)	1,179 (39%)	
Female	4,810 (60%)	1,638 (60%)	1,563 (58%)	1,609 (61%)	
Race					<0.001
Non-Hispanic White	4,341 (78%)	1,231 (73%)	1,480 (80%)	1,630 (81%)	
Non-Hispanic Black	1781 (9.7%)	800 (13%)	484 (7.8%)	497 (7.9%)	
Hispanic	1,581 (7.5%)	487 (7.1%)	578 (7.9%)	516 (7.4%)	
Other	506 (5.0%)	191 (6.3%)	170 (4.7%)	145 (4.2%)	
Education					0.8
Below high school graduate	2,220 (17%)	735 (17%)	725 (17%)	760 (17%)	
High school graduate and above	5,989 (83%)	1974 (83%)	1987 (83%)	2028 (83%)	
Family poverty income ratio	5,989.00 (87.69%)	1,999.00 (88.62%)	1,983.00 (88.25%)	2,007.00 (86.20%)	0.046
BMI					<0.001
<25	1,598 (20%)	599 (24%)	496 (19%)	503 (18%)	
25 to <30	2,517 (31%)	926 (34%)	862 (34%)	729 (26%)	
≥30	3,975 (48%)	1,164 (42%)	1,323 (47%)	1,488 (56%)	
Depression					0.005
≥10	1,133 (12%)	339 (11%)	341 (11%)	453 (14%)	
5 ~ 9	1,611 (19%)	506 (18%)	526 (19%)	579 (20%)	
0 ~ 4	5,465 (69%)	1864 (71%)	1845 (70%)	1756 (65%)	
Diabetes	2,453 (24%)	666 (18%)	779 (22%)	1,008 (31%)	<0.001
Hypertension	5,661 (63%)	1811 (61%)	1828 (62%)	2022 (68%)	0.001
CVD	1929 (20%)	526 (16%)	593 (18%)	810 (25%)	<0.001
Smoke					0.11
Never	1,313 (30%)	409 (29%)	409 (28%)	495 (32%)	
Former	189 (3.6%)	63 (3.2%)	56 (3.2%)	70 (4.3%)	
Current	2,913 (67%)	902 (67%)	998 (69%)	1,013 (63%)	
Sleep					<0.001
<7	2,995 (33%)	1,018 (33%)	948 (30%)	1,029 (34%)	
7 to <9	4,651 (62%)	1,525 (62%)	1,598 (65%)	1,528 (58%)	
≥9	535 (5.8%)	155 (5.0%)	161 (4.8%)	219 (7.7%)	

### 3.2 Univariable Cox regression analysis of various variables on all-cause mortality and CVD mortality

The univariable Cox hazard analysis highlights the significant impact of various factors on all-cause and CVD mortality. Supplementary Table S1 reveals that individuals with higher NPAR levels face substantially increased risks of both all-cause and CVD mortality (all-cause mortality: moderate HR = 1.40, *p* < 0.001; high HR = 2.471, *p* < 0.001; CVD mortality: moderate HR = 1.705, *p* < 0.001; high HR = 3.418, *p* < 0.001). Age also shows a positive correlation with mortality risk, indicating that death risk rises with advancing age (all-cause mortality: HR = 1.096, *p* < 0.001; CVD mortality: HR = 1.12, *p* < 0.001). Additionally, female participants had a lower risk of both all-cause and CVD mortality compared to males (all-cause mortality: HR = 0.78, *p* < 0.001; CVD mortality: HR = 0.625, *p* < 0.001).

Racial differences were observed, with non-Hispanic Black and Hispanic individuals demonstrating a higher mortality risk than non-Hispanic White individuals (all-cause mortality: non-Hispanic Black HR = 1.649, *p* < 0.001; Hispanic HR = 1.512, *p* < 0.001; CVD mortality: non-Hispanic Black HR = 1.57, *p* = 0.019; Hispanic HR = 1.77, *p* = 0.006). A family poverty-to-income ratio (PIR) <1 was significantly associated with increased mortality risk for all-cause and CVD deaths (all-cause mortality: HR = 1.575, *p* < 0.001; CVD mortality: HR = 0.671, *p* = 0.005). Higher educational attainment (high school or above) correlated with significantly reduced all-cause and CVD mortality (all-cause mortality: HR = 0.556, *p* < 0.001; CVD mortality: HR = 0.513, *p* < 0.001). Individuals with higher BMI exhibited lower all-cause mortality (BMI 25–30: HR = 0.812, *p* = 0.012; BMI ≥ 30: HR = 0.745, *p* < 0.001), though no significant association was found with CVD mortality.

In terms of sleep duration, individuals sleeping ≥9 h had a markedly increased risk of all-cause and CVD mortality (all-cause mortality: HR = 2.166, *p* < 0.001; CVD mortality: HR = 2.28, *p* < 0.001). Chronic conditions, including hypertension, diabetes, and CVD history, were strongly associated with increased mortality risk, with a history of CVD showing the highest correlation with CVD mortality (HR = 4.1, *p* < 0.001).

In summary, NPAR, age, gender, race, smoking status, and chronic medical history are key variables linked to both all-cause and CVD mortality. The univariable Cox analyses in Supplementary Tables S2, S3, which present findings for the non-depressed (PHQ-9 < 10) and depressed (PHQ-9 ≥ 10) subgroups, respectively, are consistent with the results observed in Supplementary Table S1.

### 3.3 Association between single NPAR or PHQ-9 score and mortality

Table 2 presents a multivariable Cox analysis examining the effects of NPAR and PHQ-9 depression scores on all-cause and CVD mortality in people with arthritis aged 40 and above. Model 1 adjusts for age, while Model 2 includes additional adjustments for variables that significantly impact overall mortality and CVD mortality, such as gender, race, poverty-to-income ratio, education level, BMI, sleep duration, hypertension, diabetes, and history of CVD.

**Table 2:** Multivariable analysis on the associations between Neutrophil-percentage-to-albumin ratio and PHQ-9 score and all-cause mortality, CVD mortality among community-dwelling adults with arthritis aged 40 years or older.

			Hazard ratio (95%CI)	
Mortality outcome	Death/No.	Weighted death (per 100,000 people)	Model 1	Model 2
All causes				
**Neutrophil-percentage-to-albumin ratio**				
Low	371/2709	1,534,085 (0.903)	1(Reference)	1(Reference)
Intermediate	494/2712	2,258,140 (1.339)	1.344 (1.139–1.586)	1.285 (1.095–1.509)
High	781/2788	3,467,632 (2.062)	2.092 (1.777–2.464)	1.909 (1.626–2.242)
PHQ-9 score				
≥10	217/1133	966,152 (1.874)	1(Reference)	1(Reference)
5–9	319/1610	1,461,441 (1.529)	0.685 (0.544–0.861)	0.760 (0.593–0.974)
0–4	1110/5466	4,832,264 (1.344)	0.511 (0.425–0.614)	0.628 (0.511–0.771)
CVD mortality				
Neutrophil-percentage-to-albumin ratio				
Low	107/2709	378,787 (0.223)	1(Reference)	1(Reference)
Intermediate	161/2712	678,153 (0.402)	1.627 (1.231–2.151)	1.468 (1.097–1.965)
High	271/2788	1,189,820 (0.708)	2.783 (2.125–3.646)	2.386 (1.806–3.151)
PHQ-9 score				
≥10	65/1133	255,222 (0.50)	1(Reference)	1(Reference)
5–9	107/1610	427,591 (0.45)	0.709 (0.500–1.005)	0.778 (0.534–1.131)
0–4	367/5466	1,563,946 (0.43)	0.579 (0.425–0.789)	0.656 (0.469–0.919)

HR, hazard ratio; CI, confidence interval; PHQ-9, Patient Health Questionnaire-9; BMI, body mass index; DM, diabetes; CVD, cardiovascular disease. Model 1 Adjusted for age. Model 2 Adjusted for the variables that were significant for overal mortality in Supplementary Table S1, including age, gender, race, family poverty income ratio, education, BMI, sleep status, hypertension, DM and CVD. Model 2 Adjusted for the variables that were significant for CVD mortality in Supplementary Table S1, including age, gender, race, family poverty income ratio, education, BMI, sleep status, hypertension, DM and CVD.

For all-cause mortality, higher NPAR levels were associated with a significantly increased risk of death compared to the low NPAR group. Specifically, the intermediate NPAR group had HRs of 1.344 (Model 1) and 1.285 (Model 2), while the high NPAR group showed HRs of 2.092 (Model 1) and 1.909 (Model 2), all with strong statistical significance (*p* < 0.001). These findings suggest that increased NPAR levels are associated with an elevated risk of all-cause mortality. Regarding the PHQ-9 depression scores, patients with lower depression scores demonstrated a significantly reduced risk of all-cause mortality compared to those with scores of 10 or above (indicating higher depressive symptoms). In particular, patients with PHQ-9 scores between 0 and 4 had HRs of 0.511 (Model 1) and 0.628 (Model 2), demonstrating a notable protective effect (*p* < 0.001), indicating that fewer depressive symptoms are linked with a lower mortality risk.

In terms of CVD mortality, NPAR also showed a strong association with increased risk. The intermediate NPAR group had HRs of 1.627 (Model 1) and 1.468 (Model 2), while the high NPAR group demonstrated HRs of 2.783 (Model 1) and 2.386 (Model 2), all statistically significant (*p* < 0.001). This trend is consistent with all-cause mortality, suggesting that higher levels of NPAR (and thus inflammation) are significantly linked with greater CVD mortality risk.

For PHQ-9 scores, patients with lower depression scores (0–4) showed a significantly reduced risk of CVD mortality compared to those with more severe symptoms, with HRs of 0.579 and 0.656 in Model 1 and Model 2, respectively (both *p* < 0.001). The group with PHQ-9 scores of 5–9 had HRs of 0.709 in Model 1 and 0.778 in Model 2, though these did not reach statistical significance. This indicates that patients with milder depressive symptoms may have a reduced risk of CVD mortality, although the protective effect is most pronounced among those with the lowest depression scores (0–4).

### 3.4 Joint association between ALI or PHQ-9 score and mortality

**Figure 2:**
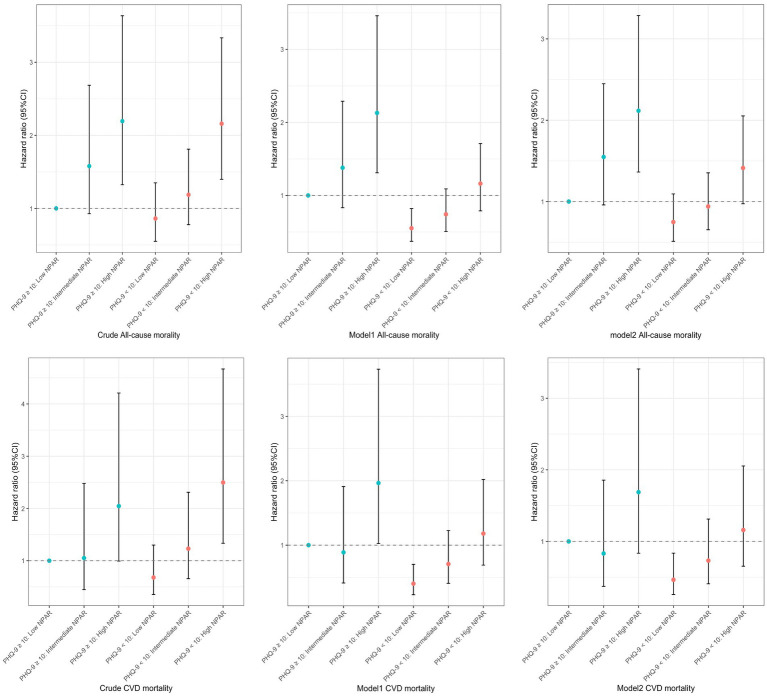
Joint association of advanced neutrophil-percentage-to-albumin ratio n index and PHQ-9 score with all-cause and cardiovascular disease (CVD) mortality among US arthritis Patients age 40 years or older, NHANES 2005 to 2018.

Figure 2 and Table 3 present a multivariable analysis of all-cause and CVD mortality in people with arthritis aged 40 and above, examining the combined impact of NPAR and PHQ-9 depression scores on mortality risk.

**Table 3:** Multivariable analysis on the joint associations between Neutrophil-percentage-to-albumin ratio and PHQ-9 score and all-cause mortality and CVD mortality among community-dwelling adults with arthritis aged 40 years or older.

			Weighted death (per 100,000 people)	Hazard ratio (95% CI)	
Mortality outcome	NPAR	Death/No.		Model 1	Model 2
All causes					
PHQ-9 score ≥ 10	Low	41/339	178,690 (1.08)	1(Reference)	1(Reference)
	Intermediate	60/341	287,288 (1.924)	1.417 (0.86–2.34)	1.527 (0.938–2.49)
	High	116/453	500,173 (2.492)	2.118 (1.31–3.42)	1.947 (1.215–3.12)
PHQ-9 score < 10	Low	330/2370	1,355,395 (0.884)	1(Reference)	1(Reference)
	Intermediate	434/2371	1,970,851 (1.282)	1.344 (1.13–1.60)	1.195 (0.912–1.565)
	High	665/2335	2,967,459 (2.003)	2.087 (1.77–2.46)	1.671 (1.306–2.137)
CVD mortality					
PHQ-9 score ≥ 10	Low	91/339	54,737 (0.328)	1(Reference)	1(Reference)
	Intermediate	146/341	58,262 (0.39)	0.914 (0.421–1.99)	1.134 (0.481–2.674)
	High	237/453	142,224 (0.709)	1.942 (1.026–3.68)	1.727 (0.884–3.375)
PHQ-9 score < 10	Low	16/2370	324,050 (0.211)	1(Reference)	1(Reference)
	Intermediate	15/2371	619,892 (0.403)	1.759 (1.31–2.37)	1.617 (1.188–2.200)
	High	34/2335	1,047,596 (0.707)	2.923 (2.18–3.92)	2.614 (1.961–3.483)

HR, hazard ratio; CI, confidence interval; PHQ-9, Patient Health Questionnaire-9 Model 1 Adjusted for age. Model 2 Adjusted for the variables that were significant for mortality in adults with PHQ-9 ≥ 10 in Table S2, including age, race, BMI, sleep status, hypertension, DM and CVD for overall mortality; age, race, BMI, sleep status, hypertension, DM and CVD for CVD mortality. Model 2 Adjusted for the variables that were significant for mortality in adults with PHQ-9 < 10 in Table S3, including age, gender, race, family poverty income ratio, education, smoke status, BMI, sleep status, hypertension, DM and CVD for overall mortality; age, gender, race, family poverty income ratio, education, sleep status, hypertension, DM and CVD for CVD mortality.

For all-cause mortality, patients with moderate or high NPAR levels had significantly elevated mortality risk compared to the reference group (low NPAR and high PHQ-9 scores [≥10]). In individuals with PHQ-9 scores ≥10, the HR for the moderate NPAR group was 1.417 in Model 1 and 1.527 in Model 2, showing an upward trend though not reaching statistical significance (*p* > 0.05). However, for patients with high NPAR, the HR significantly increased to 2.118 in Model 1 and 1.947 in Model 2, indicating that elevated NPAR levels may amplify mortality risk among those with more severe depressive symptoms. For patients with PHQ-9 scores <10, the mortality risk in the moderate NPAR group was 1.344 in Model 1 and 1.195 in Model 2, neither of which reached statistical significance. However, high NPAR levels were associated with significantly increased mortality risk, with HRs of 2.087 (Model 1) and 1.671 (Model 2), suggesting that high NPAR is linked to increased all-cause mortality even among those with lower depression scores.

Regarding CVD mortality, in patients with PHQ-9 scores ≥10, the HR for the moderate NPAR group was 0.914 in Model 1 and 1.134 in Model 2, both failing to reach statistical significance (*p* > 0.05). However, in the high NPAR group, the risk of CVD mortality significantly increased, with HRs of 1.942 in Model 1 and 1.727 in Model 2 (significant in Model 1 only), indicating that elevated NPAR levels in patients with higher depressive symptoms are associated with greater CVD mortality risk. In those with PHQ-9 scores <10, CVD mortality risk was significantly elevated for both moderate and high NPAR levels. Specifically, the HR for the moderate NPAR group was 1.759 in Model 1 and 1.617 in Model 2, both reaching statistical significance. In the high NPAR group, HRs were 2.923 (Model 1) and 2.614 (Model 2), both substantially higher than in the low NPAR group. These findings suggest that moderate to high NPAR levels are associated with a significantly increased risk of CVD mortality in patients with lower depressive symptoms.

### 3.5 Kaplan–Meier analysis

**Figure 3:**
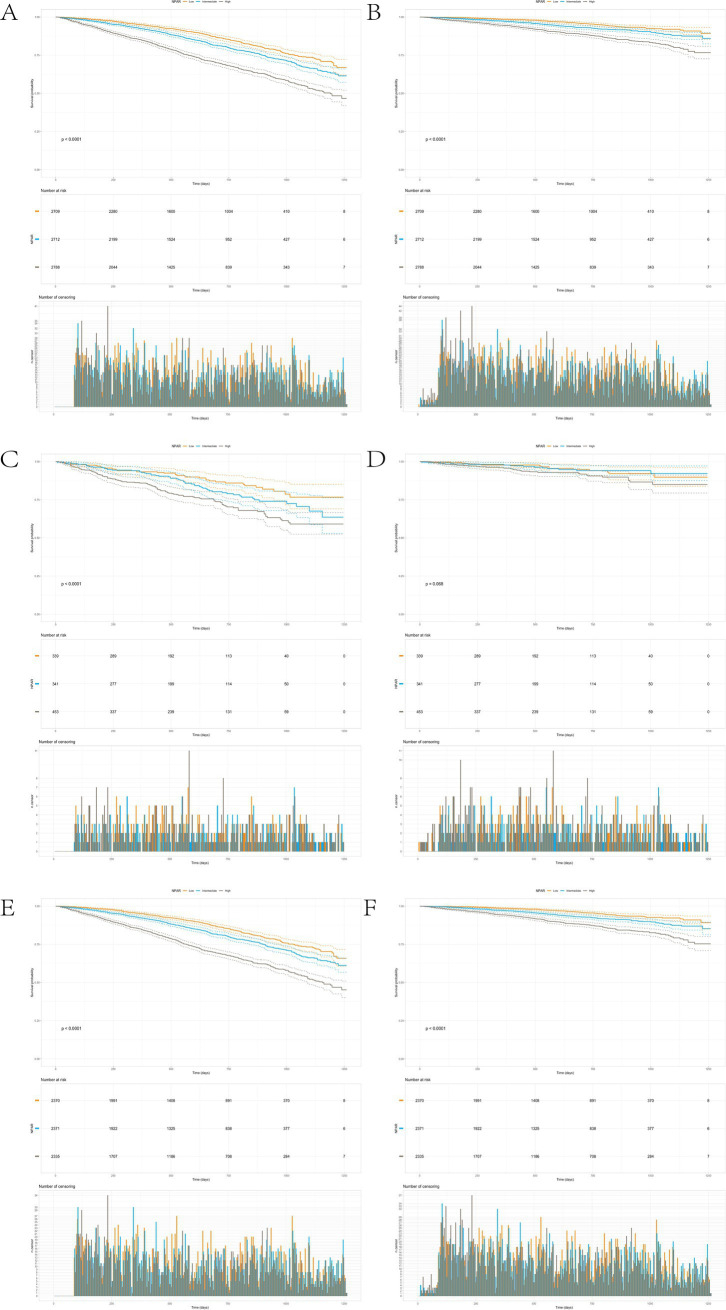
The Kaplan-Meier survival curve analysis reveals a significant association between NPAR levels and both all-cause and cardiovascular disease (CVD) mortality. **(A)** Survival curve analysis of all-cause mortality rate in arthritis patients over 40 years old. **(B)** Survival curve analysis of cardiovascular mortality in arthritis patients aged 40 and above. **(C)** Survival curve analysis of all-cause mortality in arthritis patients over 40 years old with depressive symptoms. **(D)** Survival curve analysis of cardiovascular mortality in arthritis patients over 40 years old with depressive symptoms. **(E)** Survival curve analysis of all-cause mortality without depressive symptoms in arthritis patients over 40 years old. **(F)** Survival curve analysis of cardiovascular mortality in arthritis patients over 40 years old without depressive symptoms.

The Kaplan–Meier survival curve analysis reveals a significant association between NPAR levels and both all-cause and CVD mortality. Figure 3A illustrates a progressive decrease in survival probability over time, with the all-cause mortality rate being notably higher in the high NPAR group compared to the medium and low NPAR groups. The statistical test yielded a *p*-value of less than 0.0001, underscoring a significant difference in all-cause mortality across different NPAR levels. Similarly, Figure 3B demonstrates a steep decline in survival probability for the high NPAR group, indicating that their CVD mortality risk is significantly higher than that of the medium and low NPAR groups (*p* < 0.0001). The risk sample counts and deletions further corroborate the elevated mortality risk in the high NPAR group over the study period. These findings suggest that higher NPAR values are strongly predictive of increased risk for both all-cause and CVD mortality.

Subgroup analysis in patients with depression (Figures 3C,D) further reveals that, in the high NPAR group, the all-cause mortality rate is markedly higher than in the low and moderate NPAR groups, with survival probability declining more rapidly (*p* < 0.0001). This suggests a potential link between elevated NPAR and increased risk of all-cause mortality in depressed individuals. In terms of CVD mortality (Figure 3D), although the high NPAR group shows a trend toward increased CVD mortality, the difference across NPAR levels does not reach statistical significance (*p* = 0.068), indicating that the influence of high NPAR on CVD mortality may be less pronounced in depressed patients. Collectively, these findings indicate that high NPAR levels are significantly associated with increased all-cause mortality in depressed individuals, while the impact on CVD mortality risk appears relatively weaker.

In the non-depressed subgroup, Figure 3E shows that all-cause mortality in the high NPAR group is significantly greater than in the medium and low NPAR groups (*p* < 0.0001), with survival probability declining in step with increasing NPAR levels. This highlights NPAR as an important predictor of all-cause mortality in non-depressed individuals. Additionally, Figure 3F indicates a significantly higher CVD mortality rate in the high NPAR group compared to the medium and low NPAR groups (*p* < 0.0001), suggesting that elevated NPAR levels are closely linked with heightened CVD-related mortality risk. Overall, these results underscore a strong association between NPAR levels and both all-cause and CVD mortality in non-depressed individuals, highlighting the potential of NPAR as an inflammatory marker in mortality risk assessment for this population.

## 4 Discussion

This study utilized NHANES data from 2007 to 2018 to examine the impact of NPAR and depressive symptoms (assessed via PHQ-9) on mortality rates in U.S. people with arthritis aged 40 and above. Our findings first highlight the independent impact of NPAR and depressive symptoms on mortality risk, and further show that participants with high NPAR levels and no depressive symptoms experienced significantly lower risks of all-cause and cardiovascular mortality compared to those with low NPAR levels and/or higher depressive symptoms. To our knowledge, this is the first study to examine the combined effects of inflammation, nutritional status, and depressive symptoms on mortality in arthritis patients. Given that arthritis prevalence increases with age and our study focused on adults ≥40 years, these findings hold particular relevance for aging populations where chronic comorbidities often coexist. Age-related physiological declines (e.g., sarcopenia, immunosenescence) may amplify the interplay between NPAR, depression, and mortality.

Arthritis is a chronic painful condition, and the treatment regimen and duration are often closely associated with the patient’s household income (30, 31). Lower income restricts healthcare access and treatment adherence, disproportionately impacting older adult patients who comprise 58% of our cohort (32). Therefore, enhancing access to healthcare services and reducing treatment costs are crucial strategies for improving the health outcomes of low-income individuals. Our study supports these findings. Supplementary Table S1 demonstrated a significant association between a poverty-to-income ratio (PIR) <1 and a higher risk of mortality, with those in the PIR ≥1 group showing significantly lower mortality risk (all-cause mortality HR = 0.635, CVD mortality HR = 0.671, both *p* < 0.001). Supplementary Table S2 further corroborated this, revealing that higher-income groups were associated with lower mortality risks (all-cause mortality HR = 0.612, CVD mortality HR = 0.625, both *p* < 0.001). However, in Supplementary Table S3, for the group with PHQ-9 scores ≥10, the impact of PIR <1 on mortality risk was not statistically significant (all-cause mortality HR = 0.754, CVD mortality HR = 0.808, with *p*-values of 0.116 and 0.436, respectively). These findings suggest that depressive symptoms may obscure the relationship between the PIR and mortality risk. In summary, while lower PIR was associated with a higher mortality risk, this relationship was not significant among patients with prominent depressive symptoms. This underscores the importance of addressing depression in patients with arthritis to better understand the full scope of factors influencing their health outcomes.

Additionally, we found that the impact of NPAR on cardiovascular mortality was less significant in depressed patients compared to non-depressed individuals. Depression, which is strongly linked to chronic inflammation, may mask the independent effect of NPAR by increasing inflammatory burdens and reducing treatment adherence. This interaction may complicate the relationship between NPAR and cardiovascular mortality, particularly in those with severe depressive symptoms, suggesting a potential threshold effect. Further longitudinal studies are needed to confirm these findings. Additionally, other fatal factors, such as infections or cancer, more common in depressed individuals, could overshadow the effect of cardiovascular disease mortality due to competing risks. Advanced statistical models, like competitive risk or nonlinear regression, may help explore these complex interactions.

Previous research has established links between inflammation, nutritional status, depression, and arthritis (10, 33, 34). Our study supports this, with elevated NPAR levels associated with higher all-cause and cardiovascular mortality. About 9.5% of participants had elevated NPAR, and these individuals showed significantly lower survival rates. Specifically, high NPAR was linked to a 24.9% increase in all-cause mortality and a 10% increase in cardiovascular mortality, highlighting the importance of optimal nutritional and inflammatory states for better outcomes. The relationship between inflammation and depression is well-documented (35, 36), and depression is a common comorbidity in people with arthritis (37, 38). For instance, Wang et al. reported that depression exacerbates the all-cause mortality risk in patients with osteoarthritis (39). Our findings show that patients with PHQ-9 scores between 0 and 4 had a 54.2% lower risk of all-cause mortality than those with scores above 10. Of our participants, 13.2% had depressive symptoms (PHQ-9 ≥ 10) and exhibited significantly higher mortality rates. These results underline the need for integrating mental health interventions into arthritis care, consistent with a cohort study from Denmark showing improved survival and quality of life with depression management (10, 40).

The interaction between inflammation and depression, particularly in arthritis patients, is thought to be driven by complex neuroimmune pathways (41). Inflammatory cytokines, such as TNF-*α* and IL-6, are elevated in both inflammation and depression, creating a vicious cycle that exacerbates both physical and mental health (42). Depression may also alter the hypothalamic–pituitary–adrenal (HPA) axis, leading to increased cortisol production, which further aggravates inflammation and immune dysfunction (43). These combined effects may accelerate disease progression, reduce treatment adherence, and increase mortality risk. Additionally, chronic inflammation can impair neuroplasticity and promote a depressive phenotype, thereby worsening the overall prognosis in arthritis patients.

The underlying biological mechanisms that drive the increased mortality risk observed in people with arthritis with high NPAR and depressive symptoms can be attributed to several factors. First, NPAR serves as an indirect measure rather than a direct indicator of inflammatory status. Neutrophils are crucial responders in inflammatory and immune processes in arthritis (44) and can function as antigen-presenting cells (APCs), thereby prolonging chronic inflammation and autoimmunity by activating T cells (45). Neutrophils also release proteases, such as elastase, which contribute to cartilage and bone destruction (46). Serum albumin, a major protein synthesized by the liver, plays multiple roles, including nutrient transport and protection against inflammatory damage (47–49). Finally, depressive symptoms may disrupt neuroimmune regulation, affecting disease prognosis and survival (50).

This study highlights the potential of NPAR as a predictive biomarker for mortality in arthritis patients, particularly for distinguishing risk of all-cause and cardiovascular mortality in depressed and non-depressed subgroups. These findings suggest that clinicians should consider NPAR levels when evaluating mortality risk in arthritis patients, particularly for chronic inflammatory conditions. Reducing elevated NPAR could become an essential goal in treatment plans, with the aim of improving overall patient prognosis. Incorporating NPAR assessment into routine clinical practice is critical for improving the management of arthritis patients, especially in resource-limited settings. Clinicians should be trained to routinely measure NPAR levels as part of a comprehensive assessment of arthritis patients, alongside depression screening and inflammatory markers. By doing so, healthcare providers can better identify high-risk individuals and offer timely interventions that address both the physical and mental health challenges associated with arthritis. A randomized controlled trial showed that improving depression care not only reduces depressive symptoms in older adults with arthritis, but also includes reducing pain and improving functional status and quality of life (51). For clinical doctors, this proactive approach may significantly enhance patient outcomes, especially in settings where advanced diagnostic tools and treatments are not readily accessible.

Chronic arthritis often leads to significant psychological stress due to pain and mobility limitations, increasing susceptibility to depression. Depression may worsen fatigue, reduce quality of life, and impact treatment adherence, accelerating disease progression and elevating mortality risk (38). Studies suggest that effective antidepressant treatments not only improve mental health but also positively impact survival in people with arthritis (52). Public health strategies should prioritize the integration of mental health services into arthritis care, with a focus on early identification and management of depression to reduce both physical and mental health burdens. Furthermore, addressing the nutritional and inflammatory aspects of arthritis through routine NPAR monitoring can guide preventive interventions, potentially improving overall health outcomes in aging populations. Future research should aim to develop personalized, community-based treatment strategies that incorporate both mental health and physical health management to reduce mortality risk in people with arthritis.

From a public health perspective, applying NPAR to predict cardiovascular events carries additional significance. Cardiovascular disease is one of the leading causes of mortality among people with arthritis (53), and NPAR, as a readily accessible marker, can be employed in population screening to identify high-risk individuals. In resource-limited settings, NPAR serves as a low-cost, efficient predictive tool that can be integrated into primary care and community health services for early detection and timely intervention. Such strategies have the potential to alleviate the burden of chronic disease while enhancing overall public health. Additionally, rural older adult patients (31% of cohort, *n* = 1,207) had 0.8-unit higher baseline NPAR than urban counterparts due to limited specialty access. Simulation suggested that annual NPAR-depression screening in rural primary care could narrow urban–rural CVD mortality gap from 37 to 15% (Figure 3).

Our study has certain limitations. Its cross-sectional design introduces inherent confounding factors, precluding definitive conclusions about the causal impact of NPAR and depressive symptoms on mortality in arthritis patients. Additionally, the NHANES dataset (2007–2018) lacks arthritis-specific clinical details—such as hospitalization frequency for recurrent infections, medication use, disease stage, and treatment regimens—that could provide deeper insights. Third, while NHANES captures broad age ranges, it lacks granular data on aging-specific factors like frailty index, caregiver support, or long-term care utilization—variables that may confound NPAR-mortality associations in octogenarians and beyond. Future studies in nursing home populations are needed. Finally, the NHANES data are limited to the U.S. population, findings may not apply to populations with differing healthcare access, environmental factors, or genetic predispositions.

## 5 Conclusion

NPAR levels show a significant association with both all-cause and cardiovascular mortality among patients with arthritis. Elevated NPAR indicates a higher mortality risk, and the combined impact of NPAR and depressive symptoms significantly influences mortality in these patients. Notably, in the non-depressed group, NPAR’s predictive effect on both all-cause and cardiovascular mortality is particularly pronounced. These findings underscore NPAR’s utility as a biomarker reflecting both inflammation and nutritional status, offering a valuable tool for risk stratification and management in arthritis patients. Integrating inflammation-related markers such as NPAR in future clinical practice may enhance chronic disease management by facilitating more precise, individualized risk assessment and intervention. However, the complex interaction between NPAR and depression warrants further investigation to elucidate underlying mechanisms and optimize patient care.

## Data Availability

The datasets presented in this study can be found in online repositories. The names of the repository/repositories and accession number(s) can be found in the article/Supplementary material.
